# Beyond a solvent: the roles of 1-butyl-3-methylimidazolium chloride in the acid-catalysis for cellulose depolymerisation†
†Electronic supplementary information (ESI) available: Absolute reaction rates. See DOI: 10.1039/c5sc00393h



**DOI:** 10.1039/c5sc00393h

**Published:** 2015-06-15

**Authors:** Heitor Fernando Nunes de Oliveira, Christophe Farès, Roberto Rinaldi

**Affiliations:** a Max-Planck-Institut für Kohlenforschung , Kaiser-Wilhelm-Platz 1 , 45470 , Mülheim an der Ruhr , Germany; b Imperial College London , Department of Chemical Engineering , South Kensington Campus , SW7 AZ2 London , UK . Email: rrinaldi@imperial.ac.uk

## Abstract

In this report, 1-butyl-3-methylimidazolium chloride ([C_4_C_1_im]Cl) is demonstrated to enhance the kinetics of acid-catalysed hydrolysis of 1,4-β-glucans in binary solvent mixtures. [C_4_C_1_im]Cl plays other roles in the reaction beyond acting as a solvent for cellulose, as currently accepted. In fact, the presence of the IL increases the Hammett acidity of the catalyst dissolved in the reaction medium. The kinetic data from cellobiose and cellulose hydrolysis directly correlate with the acid strength found for *p*-toluenesulfonic acid in the different reaction media studied here. The current report identifies neglected, but yet very important phenomena occurring in cellulose depolymerisation.

## Abstract 2

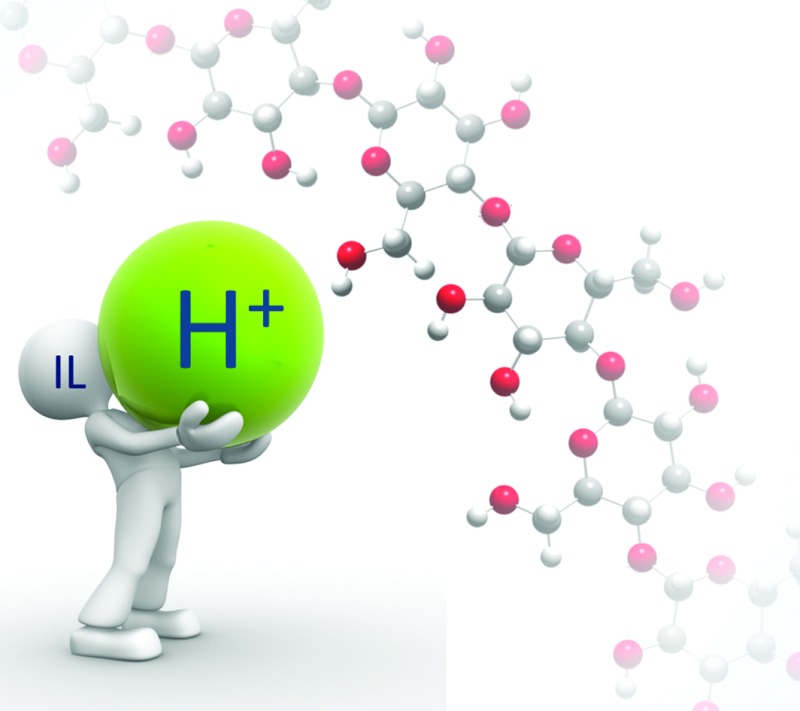
1-Butyl-3-methylimidazolium chloride plays other roles in the acid-catalysed depolymerisation of cellulose rather than being ‘merely’ a solvent for the biopolymer. The ionic liquid species enhances the Hammett acidity of the catalyst, thus improving the kinetics of cellulose depolymerisation.

## Introduction

In 2002, Rogers *et al.* introduced 1,3-dialkylimidazolium chlorides as a new class of solvents for cellulose.^1^ This breakthrough opened new avenues in cellulose chemistry, and lignocellulose deconstruction and valorisation.^2–13^ In solution, the reactivity of cellulose is markedly enhanced.^14–16^ In fact, hydrolytic processes performed in ionic liquids (ILs) proved to be highly effective, enabling cellulose to undergo hydrolysis at increased reaction rates even at temperatures as low as 100 °C.^17–21^ Currently, it is unquestioningly accepted that the enhanced accessibility of cellulose to water and molecular acid catalysts is the factor accounting for the improved reactivity of cellulose in ILs. Although the consideration on cellulose accessibility holds true, the fact that cellulose undergoes acid-catalysed hydrolysis through a mechanism involving a carbocation^22^ brings up questions whether ions could have other roles in the reaction kinetics.^23,24^

In 2011, we reported that cellulose is soluble in binary solvent mixtures comprising a minor mole fraction of a cellulose-dissolving IL in a molecular solvent (*e.g.*, DMSO).^25^ This feature enables the solvent effects of ionic liquids (ILs) upon the kinetics of cellulose depolymerisation to be studied in unprecedented detail. This is because the quantity of IL in the binary solvent mixtures can broadly be varied while the biopolymer remains in solution. Hence, it becomes possible to assess whether an IL plays other roles in the reactivity of dissolved cellulose towards acid-catalysed hydrolysis. Surprisingly, however, there is little evidence to support other roles that 1-butyl-3-methylimidazolium chloride ([C_4_C_1_im]Cl) or other chloride-based ILs may play in cellulose depolymerisation rather than be ‘merely’ a solvent.

In fact, there is evidence to suggest that the reaction rate of cellulose hydrolysis increases with *χ*_IL_ in [C_4_C_1_im]Cl/*N*-methylpyrrolidinone (NMP) mixtures.^26^ However, the chemical equilibrium NMP + H_2_SO_4_ ⇌ [NMP]H^+^ + HSO_4_^–^ was proposed to account for scavenging the acid-catalyst, and therefore, for the low hydrolysis rates obtained at low *χ*_IL_. In fact, [NMP]H^+^ shows p*K*_a_ values between –0.92 (ref. 27) and –0.17,^28^ *i.e.* about 6–34 times lower than the *K*_a_ value of H_3_O^+^ (p*K*_a_ –1.7), respectively. Hence, it is clear from that report^26^ that [C_4_C_1_im]Cl would not play itself any other role in the reaction, apart from being a (bystander) component of solvent mixture. Logically, when studying the effect of IL upon acid-catalysed reactions which require strong acids, such as cellulose hydrolysis,^29^ the best approach consists in selecting a binary solvent mixture in which both of the solvent components and H^+^ species do not react, avoiding the formation of a weak conjugated acid, and thus, an undesired decrease in the strength of the acid catalyst.^29^ Unfortunately, only a few ILs can both dissolve cellulose and be compatible with acid hydrolysis. In fact, the best ILs for cellulose dissolution comprise basic anions (*e.g.* acetate or phosphonates).^10,30,31^ These anions establish a buffer system with H_3_O^+^, drastically reducing the catalytic activity.^18^ Therefore, chloride-based (neutral) ILs still constitute the media of choice for cellulose depolymerisation performed in solution.

The understanding of solvent effects on organic reactions of interest for biomass conversion constitutes an emerging research field with potential for far-reaching applications.^32–34^ Very recently, Dumesic *et al.* ^35^ put forward a plausible explanation for solvent effects on the dehydration of xylose to furfural and hydrolysis of cellobiose to glucose in acid-catalysed reactions carried out in H_2_O and H_2_O/γ-valerolactone (H_2_O/GLV) solutions. Both reactions showed better performance in H_2_O/GLV than in H_2_O. The authors proposed that GVL affects the reaction kinetics by changing the stabilisation of the acidic proton relative to the protonated transition state.^35^ In spite of this, it is also important to bear in mind that solvents may exert multifarious effects not only on the microscopic environment of proton solvation, but also on the structural conformation of the reactants and transition states in addition to the distribution of anomers and sugar species in solution.^36^

In this report, we provide an in-depth analysis of the effects of [C_4_C_1_im]Cl on the hydrolysis of cellobiose and cellulose carried out in binary solvent mixtures of [C_4_C_1_im]Cl/dimethyl sulfoxide (DMSO). We show the effects of [C_4_C_1_im]Cl on this reaction to go beyond swelling or dissolution of cellulose, as currently accepted. This paper is organised as follow. First, the results characterising the promoting effect of ionic liquids on the hydrolysis of 1,4-β-glucans are presented. Thereafter, we address the effect of ionic liquids on the conformation of the glycosidic bond in cellobiose based on NMR long-range scalar couplings (^3^ *J*_CH_). Finally, we address the role of solvent composition in the acid strength of the catalyst dissolved in reaction media.

## Results and discussion

### Effect of [C_4_C_1_im]Cl on cellulose depolymerisation

Because of the statistical nature of the hydrolytic process, rather than the high-yield production of glucose, a decrease in the degree of polymerisation (DP) is observed in the early stages of cellulose hydrolysis.^18^ In fact, the formation of glucose requires the specific cleavage at the chain ends, while depolymerisation of cellulose can be achieved by cleaving the polymer at any position of the chain.^18,37^

**Fig. 1:**
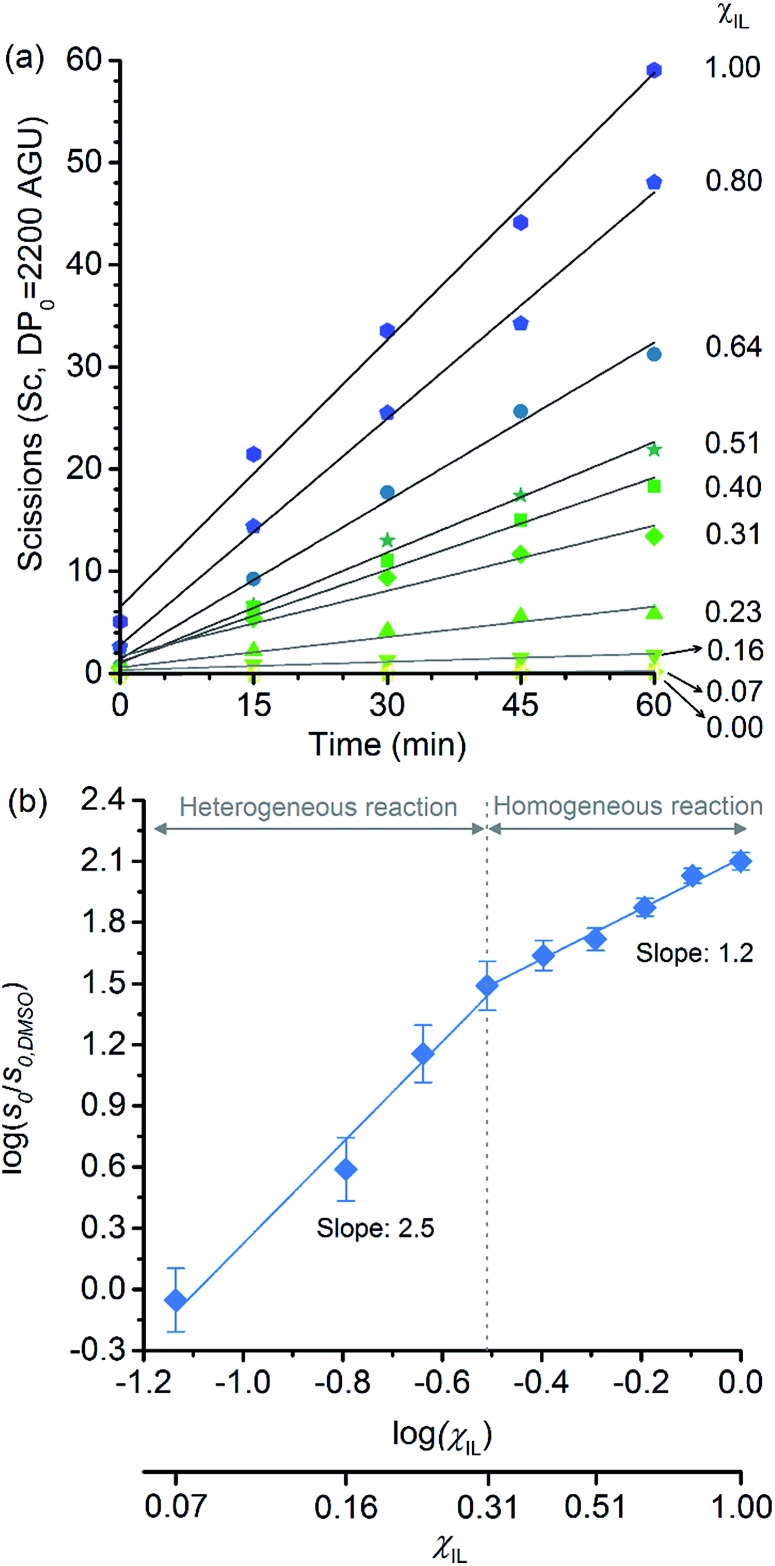
(a) Temporal evolution of the number of scissions occurring in the cellulosic chains for the experiment carried out in DMSO, [C_4_C_1_im]Cl, and [C_4_C_1_im]Cl/DMSO; (b) correlation between relative initial reaction rate (*s*_0_/*s*_0,DMSO_) and [C_4_C_1_im]Cl molar fraction (*χ*_IL_). Reaction conditions: α-cellulose (2 g, 12.4 mmol as C_6_H_10_O_5_) dispersed or dissolved in the respective solvent or binary solvent mixture (100 g), *p*-TSA (1 mmol), water (111 mmol), 100 °C. The absolute values of initial rates are available in ESI.†

The initial rates of cellulose depolymerisation can be obtained from the temporal evolution of the number of scissions occurring in the cellulosic chain. Fig. 1a shows the dependence of the temporal evolution of cellulose depolymerisation with the *χ*_IL_ present in the reaction medium. Clearly, the rate of cellulose depolymerisation increases with *χ*_IL_ in the entire range of composition of the binary solvent mixture. As a guide for the eyes in the log–log plot of the relative initial reaction rates *s*_0_/*s*_0,DMSO_ *versus χ*_IL_, two upward linear trends were drawn. For the range of 0 < *χ*_IL_ < 0.31 in which cellulose is not dissolved in the medium (*i.e.* heterogeneous reaction), a linear trend showing a slope value of 2.5 was found. The enhancement of *s*_0_/*s*_0,DMSO_ with *χ*_IL_ is commonly attributed to swelling, and then dissolution of the biopolymer (*χ*_IL_ = 0.31). As well known, the swelling and dissolution greatly improve the accessibility of cellulose to reactants. Surprisingly, however, is the fact that *s*_0_/*s*_0,DMSO_ still continues to steadily increase with *χ*_IL_ even when cellulose becomes soluble in the reaction medium (homogeneous reaction). For *χ*_IL_ > 0.31, the linear trend shows a lower value of slope (1.2) compared to the first linear trend (2.5). Altogether, the current observations provide compelling evidence to show that not only swelling of cellulose but also other phenomena are involved in the increase in *s*_0_/*s*_0,DMSO_ in the range of *χ*_IL_ > 0.31.

Clearly, as the experiments were performed at a temperature of 100 °C, no direct information concerning the apparent activation energy (*E*′a) for the depolymerisation of cellulose can be directly extracted from the data in Fig. 1. However, considering that the pre-exponential factor of Arrhenius equation should be approximately in the same order of magnitude for all the reaction systems, one can roughly estimate the corresponding Δ*E*′a from the ratio of initial rates. By using Arrhenius equation, the ratio of rates for the reactions carried out in [C_4_C_1_im]Cl and in DMSO (heterogeneous reaction) at a temperature of 100 °C corresponds to a hypothetical decrease in Δ*E*′a of about 15 kJ mol^–1^. This value agrees well with the experimental evidence showing that, when performed as a heterogeneous reaction, hydrolysis of cellulose has a value of *E*′a much higher than that of a reaction carried out with the polymer dissolved in a medium.^15,38^

Now considering the homogeneous reactions performed in [C_4_C_1_im]Cl/DMSO at *χ*_IL_ of 0.31 and in [C_4_C_1_im]Cl, the ratio of initial rates leads to a hypothetical Δ*E*′a of –4 kJ mol^–1^. To verify whether IL may cause this change in *E*′a is no easy task, because current experimental methods lack accuracy for it. On this subject, Kunov-Kruse *et al.* ^37^ reported a very elegant method for *in situ* monitoring of cellulose depolymerisation by ATR-FTIR spectroscopy. Despite the reduced size of the sample (15 mg), which is conducive to a uniform temperature distribution throughout the sample volume, an experimental deviation of ±4 kJ mol^–1^ was found for an *E*′a value of 96 kJ mol^–1^. Through conventional laboratory experiments performed on a multigram scale (5–100 g), limitations in stirring viscous cellulosic solutions in ILs causes considerable gradients of temperature. In such experiments, the average of the values reported for *E*′a is 105 ± 10 kJ mol^–1^.^29,38,39^

Therefore, the small difference in *E*′a of –4 kJ mol^–1^ is very difficult to be verified through experimental methods, and currently impossible to be ascertained by computational methods, which predict the energy of transition states, in the best cases, with an accuracy of 6–8 kJ mol^–1^. These considerations clearly reinforce the need for the development of more accurate experimental methods for the determination of reaction rates in viscous solutions in order to identify the role of ions in *E*′a of cellulose depolymerisation in IL-based solutions.

### Effect of [C_4_C_1_im]Cl on cellobiose hydrolysis

**Scheme 1:**
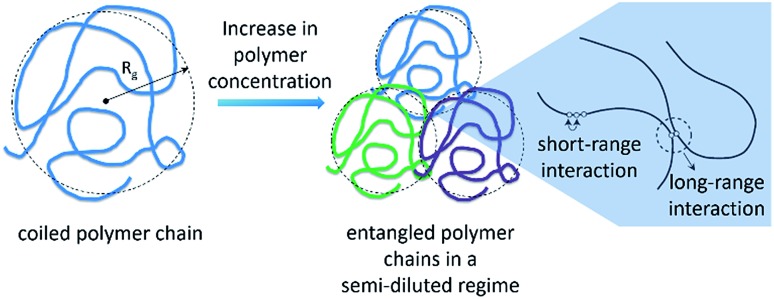
Representation of a coiled polymer chain, which is characterised by a hydrodynamic gyration radius (*R*_g_), and the entangled state of neighbouring polymer chains in a semi-diluted regime. For clarity, different colours were used in order to represent the different chains of the same polymer in the entangled state.

Another important factor to consider in the analysis of effects of IL on the depolymerisation rate is the aggregation state of cellulose in solution. It is known that macromolecules in solution form complex coiled structures (Scheme 1). In dilute solutions, the coiled structures are characterised by hydrodynamic parameters (*i.e.* gyration radius, *R*_g_, and the end-to-end distance vector).^40,41^ Logically, the solvent properties and polymer concentration affect the hydrodynamic parameters of a polymer in solution. In dilute solutions, a ‘good solvent’ is effective at dismantling the network of long-range interactions occurring between chains. Nonetheless, upon increasing the polymer concentration, neighbouring coiled polymer chains start to entangle, re-establishing other kind of long-range interactions.

Recently, Zhang *et al.* ^42^ studied the rheological properties of cellulose dissolved in binary mixtures of [C_4_C_1_im]Cl/DMSO. Interestingly, already at concentrations as low as 1.5 wt%, cellulose dissolved in [C_4_C_1_im]Cl/DMSO behaves as a typical entangled solution. Hypothetically, the entanglement of the coiled polymeric chains could limit the accessibility of cellulosic chains to reactants and molecular catalysts in the early stages of depolymerisation. The complex nature of cellulose solutions in addition to the fact that the cellulosic chains are continually converted into smaller molecules throughout the course of depolymerisation could create artefacts in the depolymerisation kinetics.

In an attempt to assess the effect of entanglement of cellulose on the depolymerisation rate, we chose to compare the effect of [C_4_C_1_im]Cl on the hydrolysis of cellobiose as a molecular model of cellulose. Unlike cellulose, cellobiose cannot become entangled. Therefore, the comparison of results from cellobiose hydrolysis and cellulose depolymerisation seems to be an adequate strategy to identify whether entanglement of cellulose could affect the reaction kinetics. Importantly, cellobiose is soluble in the entire range of the binary solvent composition, including neat DMSO, allowing for homogeneous reactions in all the media. In the reaction series for cellobiose hydrolysis, we decided to half the amount of catalyst in order to slow down the production of glucose, enabling a more accurate determination of the initial rate. Surely, reaction conditions can be tweaked in order to maximise glucose yield, as reported for cellulose hydrolysis in ILs or IL/DMSO mixtures.^21,43–45^ However, the aim of the current experiments is to determine the initial rates accurately.

**Fig. 2:**
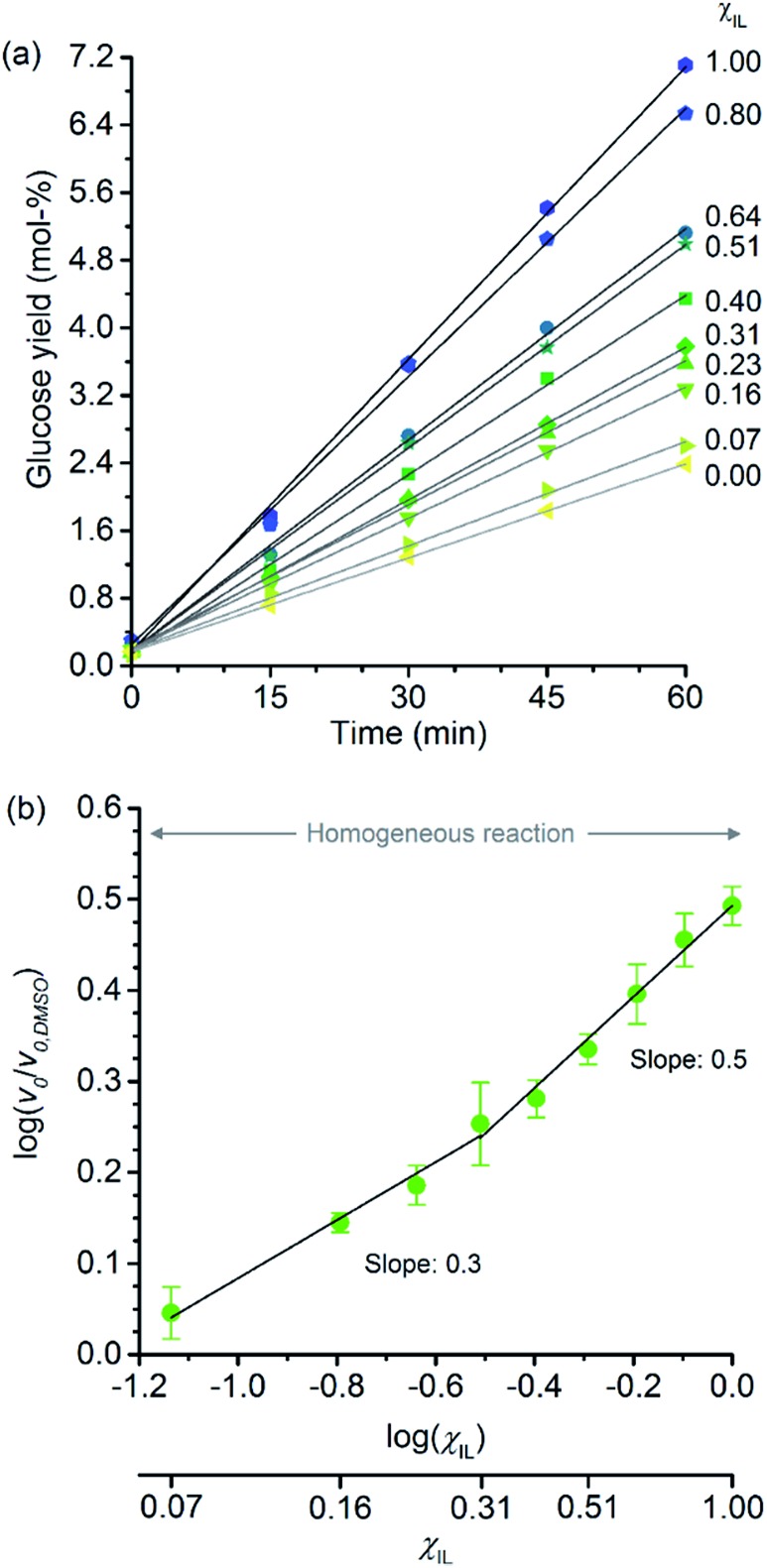
(a) Temporal monitoring of glucose formation from cellobiose hydrolysis carried out in DMSO, [C_4_C_1_im]Cl, and [C_4_C_1_im]Cl/DMSO binary solutions (b) correlation between relative initial reaction rates (*v*_0_/*v*_0,DMSO_) and mole fraction of [C_4_C_1_im]Cl (*χ*_IL_). Reaction conditions: d-(+)-cellobiose (0.58 mmol) dissolved in the respective solvent or binary solvent mixture (10 g) containing *p*-TSA (50 μmol) and water (11.1 mmol), 100 °C. The absolute values of initial rates are available in ESI.†

Fig. 2a shows the temporal evolution of glucose formation from the hydrolysis of cellobiose performed in DMSO and in the binary mixtures of [C_4_C_1_im]Cl/DMSO. Interestingly, [C_4_C_1_im]Cl accelerates the production of glucose in the entire range of *χ*_IL_. As with cellulose depolymerisation, cellobiose hydrolysis shows an increase in relative initial rates, *v*_0_/*v*_0,DMSO_, with *χ*_IL_ in the entire range of composition of the binary solvent mixtures (Fig. 2b). As a guide for the eyes in the log–log plot of the relative initial reaction rates *v*_0_/*v*_0,DMSO_ *versus χ*_IL_, two upward linear trends can be drawn in order to compare this dataset with the data presented in Fig. 1b. For the range 0< *χ*_IL_ < 0.31, a linear trend with a slope value of 0.3 was found. For *χ*_IL_ > 0.31, *v*_0_/*v*_0,DMSO_ increases with *χ*_IL_ at a higher rate, as indicated by the slope value of 0.5.

The results from cellobiose hydrolysis (Fig. 2b) reveals that the values of *v*_0_/*v*_0,DMSO_ also continuously grow with *χ*_IL_. Since cellobiose cannot become entangled, the results from Fig. 2b constitute concrete evidence that [C_4_C_1_im]Cl acts not only as a solvent but also improves the depolymerisation kinetics by other additional phenomena. Again, a hypothetical stabilisation of transition state, lowering the activation energy cannot be assessed by conventional kinetic experiments. Indeed, an eventual decrease in *E*′a, accounting for the enhancement in *v*_0_/*v*_0,DMSO_ seen in Fig. 2b, would correspond to *ca.* 3 kJ mol^–1^ (taking the ratio of reaction rates in [C_4_C_1_im]Cl and in DMSO into account).

As will be presented in the next sections, to shed light on the effects of [C_4_C_1_im]Cl on the hydrolysis of l,4-β-glucans, we examine the system from two additional perspectives. The first is to assess whether [C_4_C_1_im]Cl leads to conformational changes along the glycosidic linkage. The second perspective is to analyse how the changes in the microscopic environment of proton solvation affect the acid strength of the catalyst in the binary solvent mixtures.

### Conformational changes in the glycosidic linkage

In an attempt to elucidate the nature of [C_4_C_1_im]Cl effects on the hydrolysis of 1,4-β-glucans, our first working hypothesis was that the different solvation environments, created by varying the composition of the binary solvent mixtures, could cause conformational changes along the glycosidic linkage, thus changing the electronic structure of the acetalic system. Recently, an in-depth DFT study on the chemical nature of the 1,4-β-glycosidic bond and its chemical environment was reported.^46^ One of the conclusions from the computational predictions was that both the exo-anomeric effect (*n*_O(1)_ → σ*_C(1)O(5)_), and the intramolecular H-bonding O(5)···HO(3′) and O(2)H···O(6′), are responsible for holding the pyranic rings in a flat conformation. In this conformation, the exo-anomeric effect is maximised, and therefore, the basicity of the glycosidic O(1) site is considerably reduced. Accordingly, eventual conformation changes along the glycosidic bond, caused by the interactions with [C_4_C_1_im]Cl species, could alter the electronic effects on the acetalic system, and the chemical environment of the 1,4-β-glycosidic bond. As predicted by DFT, the basicity of the glycosidic O(1) site can be enhanced by certain changes in conformation, improving the reactivity of 1,4-β-glucans towards hydrolysis.^46^

**Scheme 2:**
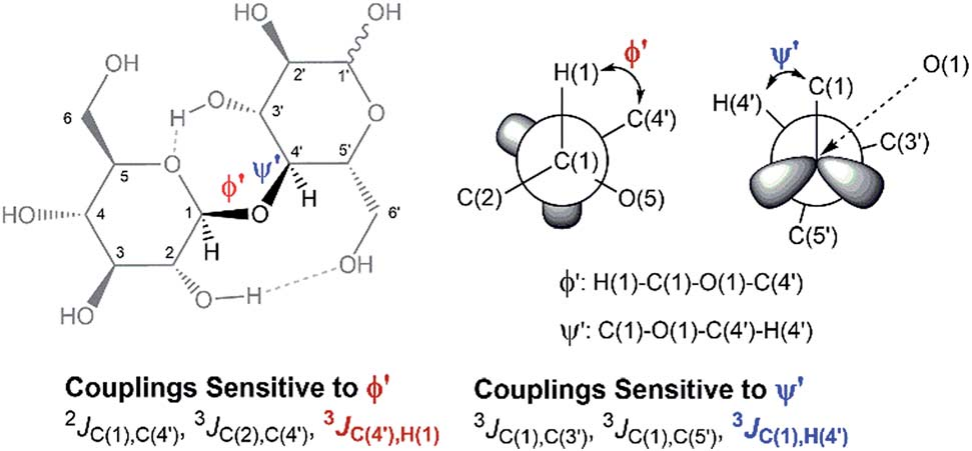
Torsion angles (*φ* ′and *Ψ*′′) across the 1,4-β-glycosidic bond and the respective *J*-couplings sensitive to *φ*′ and *Ψ*′′ according to ref. 48. The current representation of cellobiose does not correspond to an optimised structure. Dashed lines indicate the O(5)···HO(3′) and O(2)H···O(6′) intramolecular H-bonds.

To examine whether the different solvation environments alter the conformation along the 1,4-β-glycosidic bond, two key long-range scalar couplings were measured in solutions of cellobiose in selected binary perdeuterated-solvent mixtures of DMSO-d_6_ and [C_4_C_1_im]Cl-d_15_ by high resolution NMR spectroscopy using the HR-HMBC3 experiments.^47^ The multiplet pattern in this 2D heteronuclear NMR experiment can detect alterations in the vicinal scalar coupling constants ^3^ *J*_C(1),H(4′)_ and ^3^ *J*_C(4′),H(1)_ that pinpoint changes in the dihedral torsion angles *φ*′[H(1)–C(1)–O(1)–C(4′)] and *ψ*′[C(1)–O(1)–C(4′)–H(4′), as depicted in Scheme 2.^48^

**Fig. 3:**
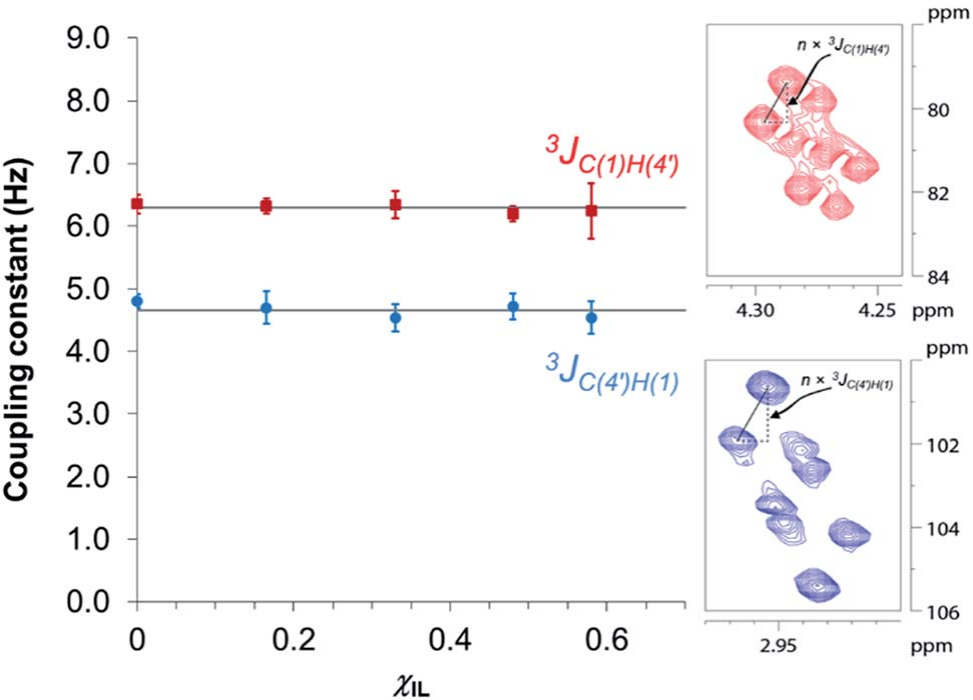
Dependence of HR-HMBC3 heteronuclear long-range coupling across the glycosidic bond with ionic liquid molar fraction. Inset: cross-peaks in the HR-HMBC3 spectrum from which the coupling was measured. The vertical displacement of peak pattern corresponds to the coupling constant value multiplied by a scaling factor (here, *n* = 25). Hence, *J*_CH_ values were obtained from the value of vertical displacement divided by 25. Solid grey lines represent the ^3^ *J*_CH_ coupling averaged values considering the coupling constants in the indicated solvent compositions (4.7 ± 0.1 Hz and 6.3 ± 0.1 Hz).

Fig. 3 reveals that no significant change in the values of ^3^ *J*_C(1)H(4′)_ and ^3^ *J*_C(4′)H(1)_ of cellobiose occurs with the increase in *χ*_IL_. This observation demonstrate that the geometry of the glycosidic bond, and therefore, the strength of this bond remains largely unaffected by the presence of [C_4_C_1_im]Cl in binary solvent mixtures with *χ*_IL_ < 0.60. Unfortunately, for cellobiose dissolved in [C_4_C_1_im]Cl-d_15_, the resonance lines were so broadened that the cross-peaks characterising ^3^ *J*_C(1),H(4′)_ and ^3^ *J*_C(4′),H(1)_ could no longer be detected in the 2D NMR spectrum. It is important to bear in mind that the increase in *χ*_IL_ substantially rise the viscosity of cellobiose solutions. The high viscosity of the solutions poses problems for the accurate determination of the vicinal scalar coupling constants even collecting the NMR spectra from samples at the temperature of 90 °C, as performed in this study. As the resonance lines become broadened, the uncertainty of the measured coupling constants increases, as seen for the determination performed on the cellobiose solution with *χ*_IL_ < 0.58.

**Fig. 4:**
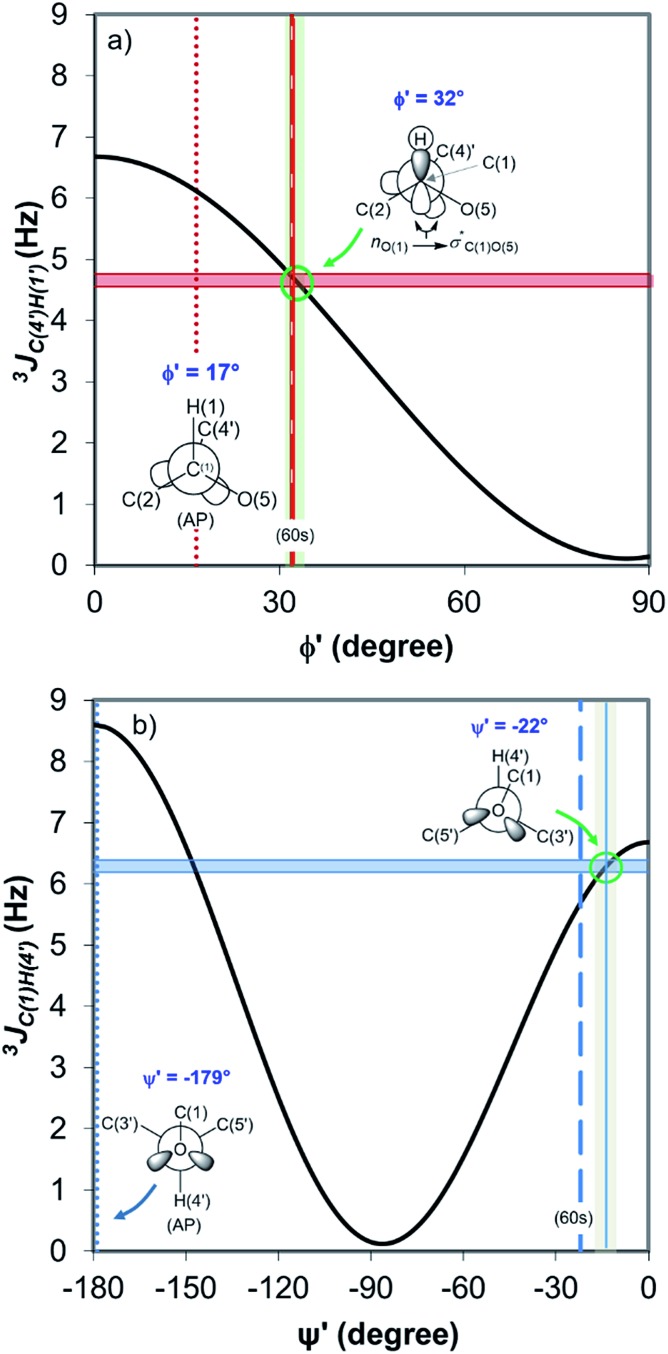
DFT-predicted Karplus curve (black) for 3-bond scalar (^3^ *J*_CH_) coupling as a function of torsion angle –C–O–C–H– according to Cloran *et al.* ^48^ Red and blue solid horizontal lines correspond to the average coupling constants, ^3^ *J*_C(4′),H(1)_ (a) and ^3^ *J*_C(1),H(4′)_ (b) measured for cellobiose in DMSO-d_6_ and [C_4_C_1_im]Cl-d_15_/DMSO-d_6_ binary solutions at 90 °C. Lowest energy torsion angles for the so-called “60 s” (dashed, [*φ*,*ψ*] = [32°,–22°]) and for the “AP” conformations (dotted, [*φ*,*ψ*] = [17°,–179°]) also represented.^50^ The measured couplings intercept the Karplus curve very close to the areas corresponding to the “60 s” conformation based on DFT calculations (green circle).^48^

Fig. 4 displays the DFT-predicted Karplus curve the for 3-bond scalar (^3^ *J*_CH_) coupling as a function of torsion angle *φ*′ (Fig. 4a) and *ψ*′ (Fig. 4b). According to the DFT-predicted Karplus relationship by Cloran *et al.*,^48^ the values for the coupling constants in cellobiose are in agreement with a conformation very close to that measured by X-ray diffraction,^49,50^ and to that predicted for the optimised geometry structure of cellobiose.^46,48^ In this conformation, the *n*_O(5)_ → σ*_C(1)O(1)_ charge transfer (exo-anomeric effect) is present (Fig. 4a), strengthening the C(1)–O(1) bond and decreasing the basicity of the glycosidic O(1) site.^46^ Alternate Karplus solutions for these coupling constants do not correspond to any possible stable rotamers of cellobiose. An alternative low-energy conformation, so-called “AP”,^48^ is also shown in Fig. 4 and does not fit with the measured couplings for cellobiose dissolved either in DMSO or in the binary solvent mixtures.

Overall, the HR-HMBC3 results reveal that [C_4_C_1_im]Cl does not affect the torsion angles of the glycosidic bond considerably. As a result, the electronic structure of the acetalic system is most likely to be the same irrespective of *χ*_IL_. Therefore, [C_4_C_1_im]Cl seems not to be involved, through induced conformational changes, in the activation of 1,4-β-glycosidic linkage towards hydrolysis.

### On the effect of [C_4_C_1_im]Cl on acid-strength of the catalyst

In earlier studies, the acid strength of H_2_SO_4_ and other acids dissolved in binary systems of molecular solvents or ILs was determined by the indicator method developed by Hammett *et al.* ^51–59^ Furthermore, this method also found use in the determination and ranking of acid strength of solid materials (*e.g.* zeolites, metal oxides, and several others).^60–62^ The acid strength is described by the extent of reaction of an acid and an indicator (a weak base, Ind), as represented by the chemical equation: Ind + H^+^ ⇌ IndH^+^. The definition of the Hammett acidity function (*H*_0_) is given by eqn (1):1
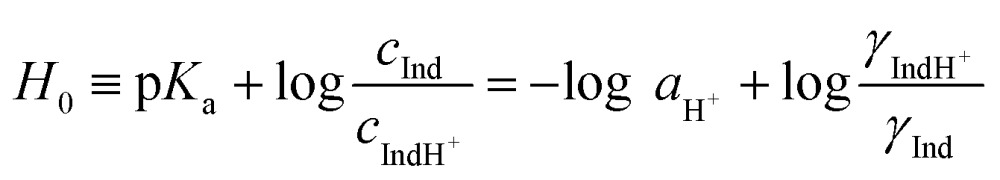
where: *c*_i_ and *γ*_i_ stand for concentration and activity coefficient of indicator (Ind) or its protonated form (IndH^+^), respectively; p*K*_a_ and *a*_H^+^_ represent the negative logarithm of the acid equilibrium constant of the protonated Hammett indicator and the proton activity in a medium, respectively. As the term log(*γ*_IndH^+^_/*γ*_Ind_) ≈ 0, the Hammett acidity function (*H*_0_) corresponds to a direct measure of acidity or the proton activity (*a*_H^+^_) in the medium.

The most common Hammett indicators are nitroaniline derivatives.^63^ In this study, we chose 4-aminoazobenzene as a Hammett indicator because the p*K*_a_ value of its protonated form (2.77) are close to the nominal acidity expected from the analytical molal concentration of *p*-TSA in the media (5 or 10 mmol kg^–1^). 4-Aminoazobenzene absorbs at the wavelength of about 405–411 nm, depending on solvent composition of the binary mixture, while its protonated form absorb visible light at around 510 nm. Hence, the extent to which the indicator is protonated can be conveniently determined on a UV-Vis spectrophotometer.

To assess whether [C_4_C_1_im]Cl exerts an effect on the acid strength of *p*-TSA dissolved in DMSO and in [C_4_C_1_im]Cl/DMSO systems, the Hammett acidity function for two concentration levels of *p*-TSA was examined. To mimic the reaction media of cellulose depolymerisation and cellobiose hydrolysis (in which water is obviously a reactant, but it also affects acidity due to the chemical equilibrium H_2_O + R–SO_3_H ⇌ H_3_O^+^ + R–SO_3_^–^), water was present in all the media at a concentration of 1.11 mol kg^–1^ (2 wt%).

**Fig. 5:**
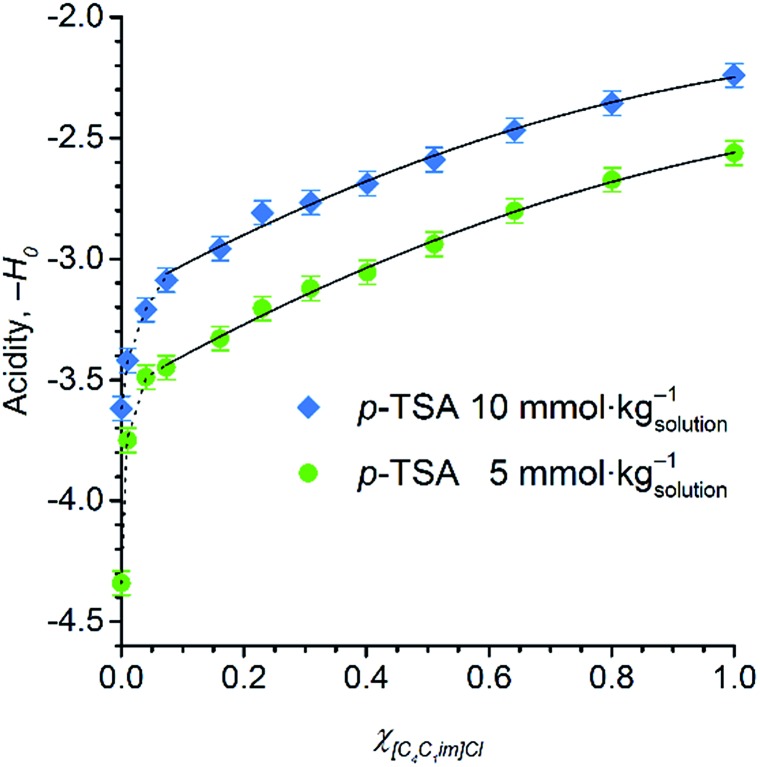
Acid strength of *p*-TSA in DMSO, [C_4_C_1_im]Cl/DMSO mixtures, and [C_4_C_1_im]Cl at 25 °C. In all the media, a 2 wt% concentration of water is present to correspond to the reaction media of cellulose depolymerisation and cellobiose hydrolysis. The self-acidity of [C_4_C_1_im]Cl/DMSO mixtures corresponds to *H*_0_ values of 4.4 ± 0.1.

Fig. 5 displays the dependence of the acidity function of *p*-TSA dissolved in DMSO and in [C_4_C_1_im]Cl/DMSO mixtures both containing 2 wt% water. For convenience, we express the acidity function as ‘–*H*_0_’ because the opposite values of *H*_0_ directly correlates to the acid strength or proton activity, since *H*_0_ ≈ –log *a*_H^+^_. For both acid-concentration levels, Fig. 5 shows that the acid strength of *p*-TSA markedly increases with *χ*_IL_. In fact, from *χ*_IL_ = 0.10 to *χ*_IL_ = 1, the apparent acid strength of *p*-TSA increased by a factor of 1.3 relative to the logarithm scale of *H*_0_ (*i.e.* about 20 times relative to *a*_H^+^_) in both datasets.

Current results indicate that the ionic species affect, indeed, the chemical equilibrium H_2_O + R–SO_3_H ⇌ H_3_O^+^ + R–SO_3_^–^. For dilute acid solutions in water, where the concentration of water is about 55 mol kg^–1^, the acidity of a strong acid (*p*-TSA, p*K*_a_ –3) is levelled out by water to the acidity of H_3_O^+^-species (p*K*_a_ –1.7). However, in the reaction media for cellulose depolymerisation or cellobiose hydrolysis, the concentration of water is 50 times lower than that of regular dilute acidic solutions in water. Moreover, computational studies on [C_4_C_1_im]Cl–H_2_O system predicted that Cl^–^ anions form a complex with three molecules of water while still keeping the H-bonding with the [BMIM]^+^ cation.^64,65^ Furthermore, water–water interactions dominates in the system only at *χ*_water_ > 0.75.^64^ Considering that *χ*_water_ is about 0.16 in our experiments, the increase in *χ*_IL_ should dramatically reduce the thermodynamic activity of water, as a substantial fraction of water molecules will be also participating in the solvation of [C_4_C_1_im]Cl species. Thus, on gradually increasing *χ*_IL_, the equilibrium H_2_O + R–SO_3_H ⇌ H_3_O^+^ + R–SO_3_^–^ is progressively shifted to the side of *p*-TSA, which shows higher acidity than H_3_O^+^. Therefore, ion solvation by water seems to mitigate levelling-out effect of water on the acidity of *p*-TSA.

**Fig. 6:**
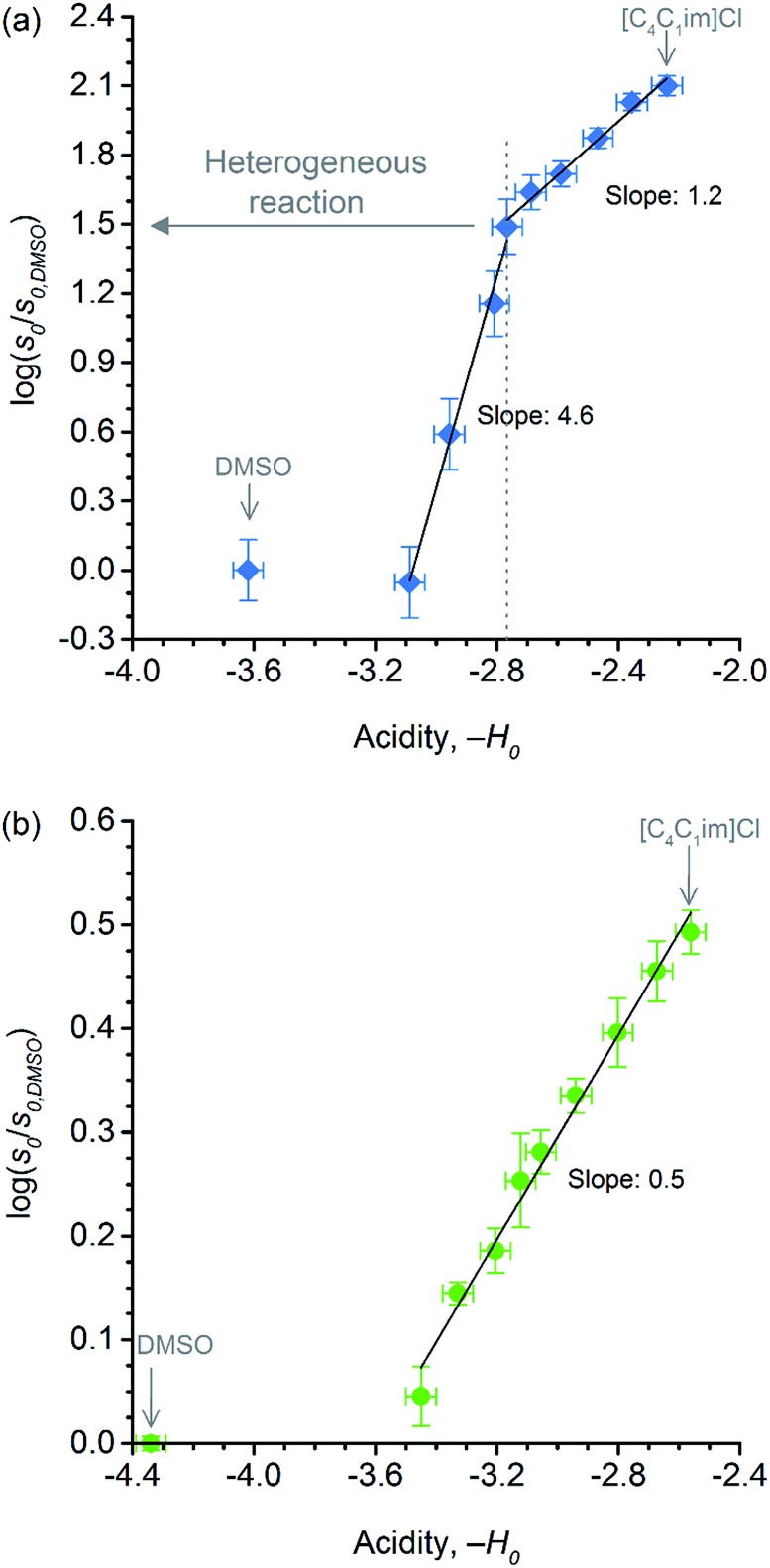
Correlations between (a) relative initial rate of cellulose depolymerisation and acidity of *p*-TSA in each reaction medium; (b) relative initial rate of cellobiose hydrolysis and acidity of *p*-TSA in each reaction medium.

Showed in Fig. 6 are the correlations between relative initial rates and –*H*_0_ values. In both datasets, the relative initial rates increase with the apparent acid strength of *p*-TSA dissolved in each reaction medium. In particular, for cellobiose hydrolysis (Fig. 6b), the excellent linear correlation between relative initial rates and –*H*_0_ demonstrates that acid strength is the key factor accounting for the kinetics enhancement in both reactions.

Indeed, the activation of 1,4-β-glycosidic bond in cellobiose and cellulose towards hydrolysis generally requires a strong acid catalyst (p*K*_a_ < –3)^18,66^ because the *n*_O(1)_ → σ*_C(1)O(5)_ charge transfer occurring in the acetalic system dramatically lowers the basicity of the O glycosidic site.^46^ For instance, in a series of monoprotonated formaldehyde acetals, R–O–CH_2_–O–R, where *R* is methyl-, ethyl- and isopropyl groups, p*K*_a_ values of –4.57, –4.13, and –3.70, respectively, were reported.^66^ Considering that the acidity of a strong acid in aqueous systems is levelled out to the acidity of H_3_O^+^-species (p*K*_a_ –1.7), the prevalence of the protonated glycosidic O sites (p*K*_a_ ∼ –4) is about 1 : 200.^18^ In [C_4_C_1_im]Cl/DMSO media, the mitigation of levelling-off effect of water, upon rising *χ*_IL_, shifts the medium acidity towards the *p*-TSA acidity (p*K*_a_ –3). Hence, the increase in the apparent acid-catalyst strength in the reaction medium leads to a larger population of protonated O glycosidic sites. As a result, the relative initial rates proportionally increase with *χ*_IL_ for both cellobiose and cellulose conversion.

Despite the effect of [C_4_C_1_im]Cl on the acid strength of *p*-TSA, the data from Fig. 6a seems to reveal another important feature. Unlike cellobiose hydrolysis, which shows only a single linear upward trend in the correlation of relative initial rate and Hammett acidity function, cellulose depolymerisation again displays two upward linear trends. In the reactions in which cellulose is not dissolved, but only swollen (first trend, *χ*_IL_ < 0.31), *χ*_IL_ determines the degree of swelling, and thus, the accessibility of cellulose to the reactants. Although both acidity and accessibility are enhanced in this range of *χ*_IL_, the accessibility seems to have a more considerable influence in the reaction rate, as revealed by the slope value of 4.6, which is about 9 times higher than that found for the series of experiments of cellobiose hydrolysis. However, even when cellulose is dissolved in the medium (*χ*_IL_ > 0.31), the effect of increasing ‘–*H*_0_’ is more pronounced on the rate of cellulose depolymerisation than that on the rate of cellobiose hydrolysis, as suggested by the slope value of 1.2, which is approximately twice as higher as that found for the data series of cellobiose hydrolysis.

The higher impact of acid strength on the kinetics of cellulose depolymerisation, relative to that on cellobiose hydrolysis, seems to suggest that the extent of protonation of O-glycosidic sites in the polymeric dispersion is greater than that in a cellobiose solution. Considering the number of short- and long-range interactions that may be established between the cellulosic chains, one could envisage that such environments could very well be solvating H_3_O^+^ species, and assisting the proton transfer mechanism.^67,68^ More importantly, in the cases that H-bonds can be established between [H^+^]-donor and [H^+^]-acceptor, the H^+^ transfer reactions are very fast and essentially diffusion controlled. Tentatively, the solvation of H_3_O^+^ species by the polymeric environment could stabilise the H^+^ species in the surroundings of the glycosidic bond. In this situation, the protonated glycosidic bond would live for a longer time,^68^ compared to that in cellobiose solution, being thus conducive to improved reaction kinetics because the protonated species must exist throughout the entire reaction coordinate, which involves conformation changes in order to activate the protonated glycosidic bond towards a heterolytic cleavage, as proposed by computational studies.^22,69^

## Conclusions

[C_4_C_1_im]Cl enhances the rate of acid-catalysed hydrolysis of cellulose and cellobiose performed in [C_4_C_1_im]Cl/DMSO solutions. The current results offer thought-provoking information on the effects of ILs upon the acid-catalysis for the hydrolysis of 1,4-β-glucans. Interestingly, 2D NMR HMBC3 experiments performed on cellobiose reveal no significant change in the values of ^3^ *J*_C(1),H(4′)_ and ^3^ *J*_C(4′),H(1)_ with the increase in *χ*_IL_. This observation shows that the geometry of the glycosidic bond remains largely unaffected by the presence of [C_4_C_1_im]Cl. Furthermore, the results provide evidence that the bond strength of the glycosidic linkage is not affected by [C_4_C_1_im]Cl. Most importantly, we found that [C_4_C_1_im]Cl enhances the Hammett acidity of *p*-TSA in the reaction media. There is a good correlation between the results from cellobiose/cellulose hydrolysis and the increase in Hammett acidity function of *p*-TSA in the reaction media. Overall, the current results bring awareness to the importance of the acid strength for the efficient hydrolysis of 1,4-β-glucans. Most importantly, these results provide evidence to demystify the misconception that the H^+^ transfer reactions is a trivial problem in the hydrolysis of 1,4-β-glucans. Clearly, to address these complex pending questions, progress in ultrafast spectroscopy is urgently needed for unravelling the intricacies of the mechanism of H^+^ transfer reactions involved in the glycosidic bond activation and cleavage.

## Experimental

### Chemicals

d-(+)-cellobiose (99.0%, Fluka), α-cellulose (cat. no. C8002, Aldrich), dimethyl sulfoxide (DMSO, ACS grade, J. T. Baker), 1-butyl-3-methylimidazolium chloride (99.999%, Iolitec), *p*-toluenesulfonic acid monohydrate (*p*-TSA·H_2_O, 98.5%, Aldrich), tetrahydrofuran (THF, 99.5%, inhibitor-free, Aldrich), methanol (MeOH, ACS grade, J. T. Baker), phenyl isocyanate (99.0%, Fluka), 4-aminoazobenzene (analytical standard, Fluka), and perdeuterated dimethyl sulfoxide (d_6_-DMSO, H_2_O < 0.02%, Eurisotop) were used as received. Perdeuterated [C_4_C_1_im]Cl ([C_4_C_1_im]Cl-d_15_) was synthesised following a synthesis protocol reported elsewhere.^70^ In the synthesis, the conventional reactants were replaced by their perdeuterated counterparts (l-methylimidazole-d_6_ and 1-chlorobutane-d_9_, Aldrich). The isotopic composition as characterised by ESI-MS is: 85% [C_4_C_1_im]Cl-d_15_ (*m*/*z* 154), 14% [C_4_C_1_im]Cl-d_14_ (in *m*/*z* 153), 1% [C_4_C_1_im]Cl-d_13_ (*m*/*z* 152).

### Acid-catalysed depolymerisation of cellulose

α-Cellulose (2 g, 12.4 mmol as C_6_H_10_O_5_) was added into a 250 mL round flask containing DMSO, [C_4_C_1_im]Cl, or a binary mixture [C_4_C_1_im]Cl/DMSO (100 g). The mixtures were heated at 100 °C for at least 1 h under mechanical stirring. α-Cellulose is soluble in the media containing *χ*_IL_ > 0.31. Nonetheless, to achieve complete homogenisation, the mixtures containing 0.31< *χ*_IL_ < 0.64 were stirred for 1 h, while those with *χ*_IL_ > 0.80 were stirred for 8 h. Into the cellulose solutions or slurries, Milli-Q water (2 mL, 111 mmol) was added under mechanical stirring. The mixture was homogenised for another additional 15 min at 100 °C. The catalytic depolymerisation was started by the addition of a 1 mol L^–1^ *p*-TSA solution in DMSO (1 mL; in the media, the final concentration of *p*-TSA was 10 mmol kg^–1^) to the mixture. The reactions were carried out at 100 °C. Aliquots (2 g) were taken throughout the experiment duration. The cellulosic fibers were regenerated from the IL-solution by addition of water (30 mL). The fibers were isolated by centrifugation, washed an additional time with water (30 mL), and finally dried overnight at 60 °C under reduced pressure (1 mbar).

### Determination of apparent degree of polymerisation

About 30 mg of recovered dry cellulose (0.2 mmol-glucan) was suspended in DMSO (5 mL). To the suspension, phenyl isocyanate (9.2 mmol) was added. The reaction was carried out for 4–5 h at 80 °C under magnetic stirring, resulting in cellulose tricarbanilates (CTC), which are soluble in DMSO. CTC was then worked up as described elsewhere.^71^ Isolated CTC was dissolved in THF (2 mg mL^–1^), and analysed by gel permeation chromatography (GPC). GPC analyses were carried out at 50 °C on a Perkin-Elmer HPLC 200 equipped with a set of mix-bed columns (2 columns, TSKgel SuperHZM-M, 4.6 mm ID × 15.0 cm) using THF as the eluent (0.2 mL min^–1^). The detection of the derivatised polymers was performed on a UV/Vis detector operating at 236 nm. The system was calibrated with polystyrene standards (5 × 10^2^ to 7 × 10^6^ Da, Aldrich). The apparent number-average and weight-average degree of polymerisation, DP_n_ and DP_w_, respectively, were calculated by dividing the corresponding average molecular weight values by the molar weight of anhydroglucose tricarbanilate (519 g mol^–1^). The equivalent number of scissions was calculated by using eqn (2):2
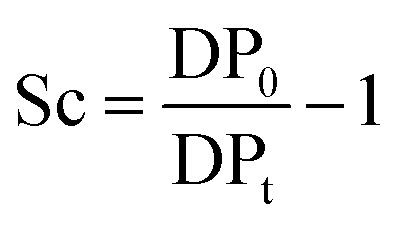
where: DP_0_ and DP_t_ stand for the weight-average degree of polymerisation determined for α-cellulose as received (DP_0_ = 2200 anhydroglucose units, AGU) and for the regenerated cellulose obtained from depolymerisation experiments at a given time, respectively.

### Hydrolysis of cellobiose

d-(+)-cellobiose (200 mg, 0.58 mmol) was added into a 20 mL vial containing DMSO, [C_4_C_1_im]Cl, or a binary mixture [C_4_C_1_im]Cl/DMSO (10 g) at 100 °C. The mixture was stirred by a magnetic stirrer for 15 min. To cellobiose solutions, Milli-Q water (200 μL, 11.1 mmol) was added under magnetic stirring. The mixture was homogenised for another additional 15 min at 100 °C. The catalytic hydrolysis was initiated by the addition of a 1 mol L^–1^ *p*-TSA solution in DMSO (50 μL; in the media, the final concentration of *p*-TSA was 5 mmol kg^–1^) to the mixture. The experiments were carried out at 100 °C. Aliquots (0.5 g) were taken throughout the experiment duration and diluted with Milli-Q water (0.5 mL) for chromatographic analysis.

### Cellobiose and glucose quantification

Cellobiose and glucose were analysed by gel filtration chromatography (GFC). Analyses were performed at 25 °C on a Perkin-Elmer HPLC 200 equipped with a set of two columns (TSKgel G-Oligo-PW: 7.8 mm I.D. × 30 cm, Tosoh), and using Milli-Q water as the eluent (1.0 mL min^–1^). The quantification of cellobiose and glucose was performed on a refractive index detector operating at 40 °C.

### High-resolution heteronuclear multiple bond correlations (2D NMR HMBC3) measurements

The NMR experiments were performed on a Bruker AVIII 500 MHz spectrometer equipped with a BBFO + probehead tuned to ^13^C (125 MHz) and ^1^H (500 MHz). The HR-HMBC3 experiment with a scaling factor of 25 was used in order to measure the heteronuclear long-range coupling across the glycosidic bond (3-bond correlations). Cellobiose samples (2 wt%) in DMSO-d_6_ and [C_4_C_1_im]Cl-d_15_/DMSO-d_6_ binary solvent mixture were filled into a 5 mm NMR tube. A narrow (2 mm) concentric glass insert containing TMS 2 vol% in DMSO-d_6_ for the purposes of locking and chemical shift reference was used. The samples were measured at 90 °C.

### Acidity of the reaction medium

UV-Vis spectra of the Hammett indicator 4-aminoazobenzene (4AAB, p*K*_a_ 2.77) in DMSO, [C_4_C_1_im]Cl, or [C_4_C_1_im]Cl/DMSO solutions in presence and in absence of *p*-TSA were recorded on an Agilent 8453 UV-Vis spectrophotometer. Typically, 20 μL of indicator stock solution in DMSO (4 mmol L^–1^) were added into a quartz cell (path length of 0.5 cm) filled with 1 mL of the medium of interest (containing 2 wt% H_2_O). Solutions with high load of IL were warmed up at 80 °C in order to guarantee complete mixing of indicator solution with medium. After homogenisation, the solution was kept at 25 °C for UV-Vis analysis. The UV-Vis spectrum of the medium with the indicator was subtracted from the corresponding blank spectrum of the medium. In absence of *p*-TSA, the maximum absorbance for 4AAB (404–411 nm) was kept always below 1.6 A.U. This procedure guarantees that the absorbance values are within the linear range of Lambert–Beer law. Eqn (3) describes how the *H*_0_ values were determined in this work. *A*_4AAB,l_ and *A*_4AAB,2_ stand for the maximum absorbance of 4AAB determined in the absence and in the presence of *p*-TSA, respectively.3
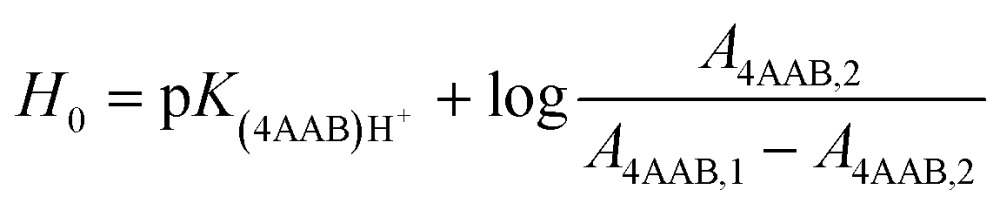
where: ‘*A*_4AAB,2_’ is proportional to the equilibrium concentration of free base (4AAB), and ‘*A*_4AAB,1_ – *A*_4AAB,2_’ to the equilibrium concentration of protonated indicator [(4AAB)H^+^].
