# Supplementary material for: Beyond a solvent: the roles of 1-butyl-3-methylimidazolium chloride in the acid-catalysis for cellulose depolymerisation
Source: Chem Sci. 2015 Jun 15;6(9):5215–24. doi: 10.1039/c5sc00393h (PMC5500853; doi:10.1039/c5sc00393h)
Supplement: Supplementary file 1 [file SC-006-C5SC00393H-s001.pdf]

Electronic Supplementary Material (ESI) for Chemical Science.
This journal is © The Royal Society of Chemistry 2015

Electronic Supplementary Information

Beyond a solvent: The roles of 1-butyl-3-methylimidazolium chloride in the acid-catalysis for cellulose depolymerisation

Heitor Fernando Nunes de Oliveira,[a] Christophe Farès,[a] Roberto Rinaldi[a,b,*]

Max-Planck-Institut für Kohlenforschung, Kaiser-Wilhelm-Platz 1, 45470, Mülheim an der Ruhr, Germany.
Imperial College London, Department of Chemical Engineering, South Kensington Campus, SW7 AZ2 London, United Kingdom. E-mail: rrinaldi@imperial.ac.uk

Table S1 Initial reaction rate for cellulose (s_0) and cellobiose (v_0) hydrolysis and acidity (H_0) of the corresponding reaction media in DMSO, [BMIM]Cl, and DMSO/[BMIM]Cl binary mixtures.

[BMIM]Cl / wt%	$\chi_{[BMIM]Cl}$	Cellulose		Cellobiose	
		s_0 / Scissions·min^{-1}	H_0 (±0.05)*	v_0 / Y_{Glu}·min^{-1}	H_0 (±0.05)*
0	0.000	0.0069	3.62	0.039	4.34
2.2	0.010	—	3.42	—	3.75
8.5	0.040	—	3.21	—	3.49
15	0.073	0.0061	3.09	0.043	3.45
30	0.161	0.027	2.96	0.054	3.33
40	0.230	0.10	2.81	0.059	3.21
50	0.309	0.21	2.77	0.069	3.12
60	0.402	0.30	2.69	0.074	3.06
70	0.511	0.36	2.59	0.084	2.94
80	0.642	0.52	2.48	0.096	2.80
90	0.801	0.74	2.36	0.110	2.67
100	1.000	0.87	2.24	0.120	2.56

* In all the media, a 2 wt% concentration of water is present to correspond to the reaction media of cellulose depolymerisation and cellobiose hydrolysis.
